# Impact of dementia on risks of COVID-19 infection and outcomes among older adults in Sweden

**DOI:** 10.1186/s12877-025-06576-3

**Published:** 2025-11-04

**Authors:** Minh Tuan Hoang, Jonas W. Wastesson, Máté Szilcz, Géric Maura, Pierre-Olivier Blotière, Kristina Johnell

**Affiliations:** 1 Department of Medical Epidemiology and Biostatistics, Karolinska Institutet, Blickagången 16, Stockholm, 14183 Sweden; 2 Department of Neurobiology, Care Sciences and Society, Division of Clinical Geriatrics, Karolinska Institutet, Stockholm, Sweden; 3 Faculty of Public Health, the University of Medicine and Pharmacy, Vietnam National University, Hanoi, Vietnam; 4 Department of Neurobiology, Care Sciences and Society, Aging Research Center, Karolinska Institutet and Stockholm University, Stockholm, Sweden

**Keywords:** COVID-19, Death, Dementia, Hospitalization, Infection, Mortality, SARS-CoV-2

## Abstract

**Background:**

National register-based studies on the influence of dementia on COVID-19 infection and outcomes are scarce. This study aims to evaluate the risk of COVID-19 infection, hospitalization, and mortality among older persons with and without dementia.

**Methods:**

This population-based observational study utilized real-world data based on the combination of various national registries in Sweden. Outcomes included COVID-19 infection, hospitalization, and mortality between 01 March 2020 (index date) and 31 August 2020, which was usually considered as the first wave of COVID-19 in Sweden. We used flexible parametric survival regression to estimate the hazard ratios (HRs) between people with and without dementia at different time points after the index date.

**Results:**

We compared people with and without dementia, who were living in community dwellings (40,818 versus 1,984,503 persons) or nursing homes (31,826 versus 87,398 persons). In community dwellings, the HRs of COVID-19 infection increased from 2.08 in one month to 2.46 in two months, then decreased to 0.70 in six months after the index date. In nursing homes, the HRs declined throughout the follow-up period (from 1.89 in one month to 0.91 in six months). In community dwellings, the HRs of COVID-19 hospitalization increased from 1.40 in one month to 1.64 in six months after the index date. In nursing homes, the HRs was less than 1 in one month, and higher than 1 from four months after the index date, however, not statistically significantly. The HRs for COVID-19 mortality rose from 1.96 in one month to 2.39 in two months and dropped to 1.65 in six months in community dwellings; and declined from 2.27 in one month to 1.69 in six months in nursing homes.

**Conclusions:**

In the first wave of COVID-19 in Sweden, higher risks of COVID-19 infection, hospitalization and mortality were observed in older persons with dementia compared to those without dementia, except for the risk of COVID-19 hospitalization in nursing homes. Further studies on the quality of care for persons with dementia are essential to prepare for future pandemics.

**Supplementary Information:**

The online version contains supplementary material available at 10.1186/s12877-025-06576-3.

## Introduction

Higher age and underlying medical conditions were the strongest risk factors of severe COVID-19 infection and mortality [1, 2]. Compared to persons aged 18–29, older persons aged 65 years old or above were at 60 to 340 times higher risk of COVID-19 mortality between 2020 and 2022 [1]. Older persons with dementia were among the most vulnerable groups during the COVID-19 pandemic, potentially due to their cognitive impairment, disability, dependence in activities of daily living, and other comorbidities [3, 4]. Yet, nationwide data on the excess risks for COVID-19 outcomes in persons living with dementia are scarce.

Previous systematic reviews and meta-analyses mentioned that the risk of COVID-19 mortality was higher among persons with dementia, compared to those without dementia [5–8]. Later observational studies in the United Kingdom and the United States also showed that persons with dementia were at higher risks of COVID-19 infections compared to persons without dementia [9–12]. In Sweden, a recent study on COVID-19 mortality among persons living in nursing homes found that dementia was associated with higher risks of COVID-19 infection and mortality [13]. Another study in nine geriatric care clinics in Stockholm, Sweden observed a higher risk of COVID-19 mortality in persons with dementia, compared to non-dementia counterparts [14].

Most of the preceding studies on dementia and COVID-19 were based on selected settings, such as hospital-based, regional scale or primary care dataset, rather than on national big data. Moreover, the differentiation between older persons living in community dwellings and those living in nursing homes was not mentioned in previous studies, although living arrangements might influence care decisions and care trajectories [15]. Using a combination of various Swedish nationwide registries with the real-world data, we aimed to explore this knowledge gap in our study.

To this end, the objective of our study was to investigate the association between dementia and the risks of COVID-19 infection, hospitalization, and mortality among older persons in Sweden. We hypothesized that older persons with dementia were at higher risks of COVID-19 infection, hospitalization, and mortality than the general older population without dementia, irrespective of living arrangements.

## Methods

### Study design and setting

This observational study was conducted on data from the Total Population Register, the Swedish Longitudinal Integrated Database for Health Insurance and Labor Market Studies (LISA), the National Patient Register, the National Prescribed Drug Register, the National Cause of Death Register, the Social Services Register and the Swedish Registry of Infectious Diseases, linked by pseudonymized Swedish personal identification numbers [16]. The description of these national registers is summarized in Supplementary eTable 1. This study focused on the first wave (01 March 2020 (index date) to 31 August 2020) of COVID-19 pandemic in Sweden [17, 18]. A study design diagram is presented in Supplementary eFigure 1.

### Participants

People with dementia were defined as persons registered with the International Classification of Diseases (ICD) 10th codes F00-F03, F051, G30, G31 in the National Patient Register or with the Anatomical Therapeutic Chemical code N06D in the National Prescribed Drug Register between 01 January 2014 and the index date. People without dementia were defined as persons not registered with these codes until the index date. We used an extensive look back period until 01 January 2014 to capture all the dementia diagnosis because the median survival time of persons with dementia is about 5 to 6 years after dementia diagnosis [19–21]. Exclusion criteria for both groups were: (1) Aged 65 years old or above at the index date, (2) migrated to Sweden after the index date, (3) had COVID-19 before the index date (Supplementary eFigure 2).

### Main exposure and covariates

The main exposure was having dementia versus not having dementia. Covariates included sociodemographic data which were extracted from the Total Population Register and LISA: age at index date, sex, living areas (rural, intermediate versus urban), cohabitation status (living alone versus cohabiting), and education. The highest attained education at the index date was categorized into compulsory education (primary school and secondary school [years 1–9]), upper secondary (high school [years 10–12]) and university (college, university or higher). Living arrangement (nursing homes versus. community dwellings) at the index date was defined based on data from the Social Service Register, which records monthly information on home care services and nursing home residence. In Sweden, admission to nursing homes generally occurs when an individual's care needs exceed what can be managed in the home setting (professionally assessed). Nursing homes provide 24-h staff presence and support with activities of daily living. Most facilities also offer basic nursing care, and access to physicians is typically arranged through municipal or regional primary care services, although on-site medical staff may vary between municipalities. Older people will mostly continue to live in nursing homes until their death. Individuals were considered as nursing home residents if they had been admitted to nursing homes at any point before the index date, and as community-dwellers if they had no record of previous nursing home residence.

Chronic comorbidities [22] and hospital frailty risk score [23] during five years before the index date were summarized with data from the National Patient Register or the Prescribed Drug Register (Supplementary eTables 2 & 3). We considered chronic comorbidities that were associated with higher risk of severe COVID-19, including asthma, atrial fibrillation, cerebrovascular diseases, chronic infectious diseases, chronic kidney diseases, chronic liver diseases, chronic obstructive pulmonary disease, depression, diabetes, heart failure, hypertension, ischemic heart diseases, obesity, osteoporosis, Parkinson and parkinsonism, and peripheral vascular diseases [1, 10]. The number of drugs used during the three months before the index date was captured and categorized at the second level of pharmacological subgroups of the Anatomical Therapeutic Chemical codes from the National Prescribed Drug Register.

### Outcomes

Outcomes included COVID-19 infection, hospitalization, and mortality. COVID-19 infection was defined as clinically confirmed COVID-19 cases by positive tests, which were captured from the Swedish Registry of Infectious Diseases. COVID-19 hospitalization was extracted from the National Patient Register with ICD 10th codes U071 (COVID-19, virus identified) and U072 (COVID-19, virus not identified). COVID-19 deaths, retrieved from the National Cause of Death Register, were identified using ICD 10th codes U071, U072 as the underlying or contributing causes of death.

### Statistical analysis

Descriptive statistics were presented with the number and percentage of individuals or with means and standard deviation. All analyses were stratified by living arrangements due to the excessive number of COVID-19 deaths in nursing homes in Sweden [24, 25]. We presented the incidence rate (IR) per 1000 person-months (95% confidence interval – 95% CI) with the underlying time scale of months after the index date. Censoring was defined as the occurrence of each outcome, or emigration, or the new dementia diagnosis (only for people without dementia), or nursing home admission (only for people living at community dwellings at the index date), or death, or termination of the follow-up (31 August 2020), whichever happened first.

Due to the violation of the proportional hazard assumption in Cox regression, we employed Royston-Parmar flexible parametric regressions for the main analyses (STATA package stpm3) [26, 27]. The flexible parametric competing-risks model was used to estimate cause-specific cumulative incidence functions and subdistribution hazard ratios of each outcome between people with and without dementia at different time points after the index date [28, 29]. All-cause mortality was the competing event when outcomes were COVID-19 infection and hospitalization. Another-cause mortality was the competing event when outcome was COVID-19 mortality. These analyses were adjusted for age, sex, living areas, cohabitation status, education, chronic diseases, hospital frailty risk score, and the number of drugs. All covariates were time-fixed and proportional hazards were assumed for these confounders. Results were presented as subdistribution hazard ratios (HR) and 95% confidence interval (95% CI) of the interested outcomes, which accounted for the possible occurrence of the competing events.

Sensitivity analysis using a propensity score matched cohort was performed to ensure that the comparison between persons with and without dementia was valid. In logistic regression models, we predicted the propensity score of having dementia based on age, sex, living areas, cohabitation status, education, chronic diseases, hospital frailty risk score, and number of drugs. Afterwards, a 1:1 propensity score matching was performed with the nearest neighbors matching method, without replacement and with a caliper of 0.01.

Statistical analysis was performed with STATA version 17.0 (StataCorp, College Station, TX). This study was reported in accordance with the REporting of studies Conducted using Observational Routinely collected health Data (RECORD) statement [30] (Supplementary eTable 4).

## Results

### Characteristics of the study cohort

In this study cohort, about 5.6% selected persons lived in a nursing home setting. About 26.7% of the persons living in nursing homes had a record of dementia, compared to 2.0% in community dwellings (Table 1). In community dwellings, people with dementia had a higher mean age at index date (81.2 versus 74.9 years old), higher proportion of female (54.4% versus 52.7%), a lower proportion of cohabiting (62.4% versus 67.1%), a higher proportion of individuals with a high hospital frailty risk score (12.9% versus 2.6%) and had more chronic comorbidities, compared to individuals without dementia. In nursing homes, the mean ages of persons with and without dementia were 85.1 and 81.8 years old, respectively. Females accounted for 65.8% and 59.4%, respectively in people with and without dementia. About 32.0% of persons with dementia and 31.5% of persons without dementia were cohabiting at index date. People with dementia had a higher proportion of high hospital frailty risk score (24.5% versus 18.2%), and higher proportions of chronic comorbidities, compared to people without dementia.

**Table 1:** Characteristics of study cohort

	**Older people living in community dwellings**		**Older people living in nursing homes**	
	*non-Dementia*	*Dementia*	*non-Dementia*	*Dementia*
N	1,984,503	40,818	87,398	31,826
Age, years, mean ± SD	74.9 ± 7.1	81.2 ± 6.8	81.8 ± 9.9	85.1 ± 7.2
Female, n (%)	1,045,863 (52.7)	22,198 (54.4)	51,922 (59.4)	20,957 (65.8)
Living areas, n (%)				
* Urban*	598,748 (30.2)	12,531 (30.7)	25,197 (28.8)	9328 (29.3)
* Intermediate*	814,911 (41.1)	15,799 (38.7)	36,438 (41.7)	12,899 (40.5)
* Rural*	570,844 (28.8)	12,488 (30.6)	25,763 (29.5)	9599 (30.2)
Cohabitation status, n (%)				
*Cohabiting*	1,330,861 (67.1)	25,475 (62.4)	27,491 (31.5)	10,192 (32.0)
*Living alone*	653,642 (32.9)	15,343 (37.6)	59,907 (68.5)	21,634 (68.0)
Education, n (%)				
*University/College*	567,595 (28.6)	9205 (22.6)	15,628 (17.9)	5582 (17.5)
*Secondary education*	827,149 (41.7)	15,985 (39.2)	31,652 (36.2)	11,584 (36.4)
*Compulsory education*	565,967 (28.5)	14,942 (36.6)	37,509 (42.9)	14,158 (44.5)
*Unknown*	23,792 (1.2)	686 (1.7)	2609 (3.0)	502 (1.6)
Hospital Frailty Risk Score, n (%)				
*Low (*< *5)*	1,523,039 (76.7)	17,614 (43.2)	38,010 (43.5)	8062 (25.3)
*Moderate (5–15)*	410,636 (20.7)	17,944 (44.0)	33,497 (38.3)	15,980 (50.2)
*High (*> *15)*	50,828 (2.6)	5260 (12.9)	15,891 (18.2)	7784 (24.5)
Number of drugs, n (%)				
< *5*	1,298,732 (65.4)	16,784 (41.1)	32,376 (37.0)	6746 (21.2)
*5–9*	574,560 (29.0)	19,478 (47.7)	37,806 (43.3)	18,783 (59.0)
≥ *10*	111,211 (5.6)	4556 (11.2)	17,216 (19.7)	6297 (19.8)
Chronic comorbidities, n (%)				
*Asthma*	79,100 (4.0)	2105 (5.2)	4931 (5.6)	1745 (5.5)
*Atrial fibrillation*	235,335 (11.9)	8140 (19.9)	19,251 (22.0)	7290 (22.9)
*Cerebrovascular diseases*	186,134 (9.4)	8617 (21.1)	21,618 (24.7)	8587 (27.0)
*Chronic infectious diseases*	17,745 (0.9)	480 (1.2)	1002 (1.1)	355 (1.1)
*Chronic kidney diseases*	55,843 (2.8)	1989 (4.9)	5200 (5.9)	1695 (5.3)
*Chronic liver diseases*	16,946 (0.9)	343 (0.8)	1405 (1.6)	230 (0.7)
*COPD*	93,829 (4.7)	2762 (6.8)	7532 (8.6)	2284 (7.2)
*Depression*	75,329 (3.8)	4170 (10.2)	9918 (11.3)	4150 (13.0)
*Diabetes*	230,949 (11.6)	7083 (17.4)	15,390 (17.6)	5784 (18.2)
*Heart failure*	133,344 (6.7)	4954 (12.1)	14,714 (16.8)	4811 (15.1)
*Hypertension*	718,540 (36.2)	21,945 (53.8)	46,697 (53.4)	19,024 (59.8)
*Ischemic heart diseases*	260,037 (13.1)	8352 (20.5)	17,717 (20.3)	6739 (21.2)
*Obesity*	52,548 (2.6)	1000 (2.4)	3091 (3.5)	641 (2.0)
*Osteoporosis*	69,822 (3.5)	2910 (7.1)	7315 (8.4)	3420 (10.7)
*Parkinson and parkinsonism*	15,740 (0.8)	2362 (5.8)	2599 (3.0)	1777 (5.6)
*Peripheral vascular diseases*	44,029 (2.2)	1337 (3.3)	4073 (4.7)	1167 (3.7)

*SD* Standard deviation, *COPD* Chronic obstructive pulmonary disease

### COVID-19 infection

**Fig. 1:**
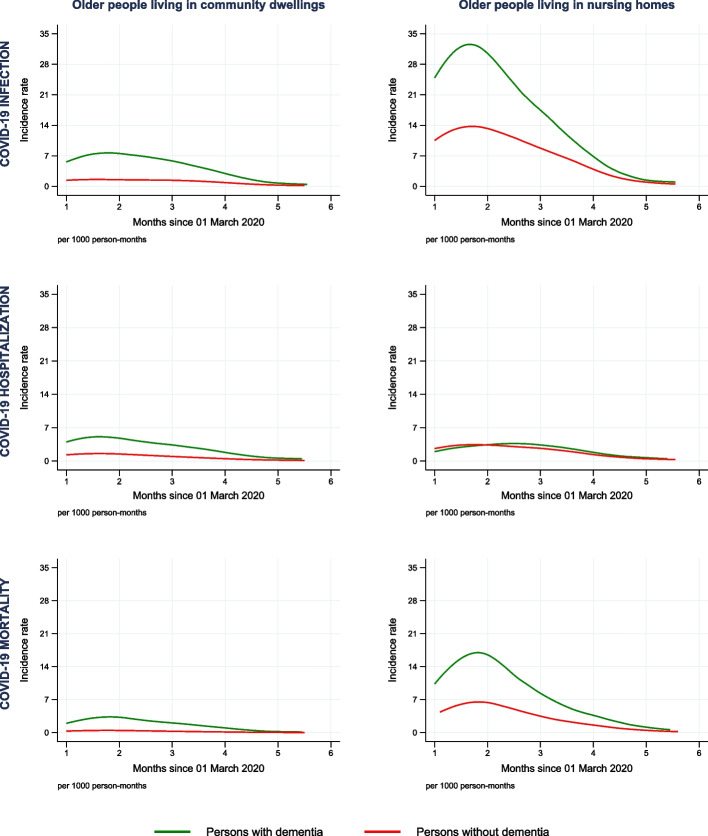
Cause-specific incidence rate of COVID-19 infection, hospitalization, and mortality between older persons with and without dementia (estimated with the flexible parametric competing-risks model)

**Fig. 2:**
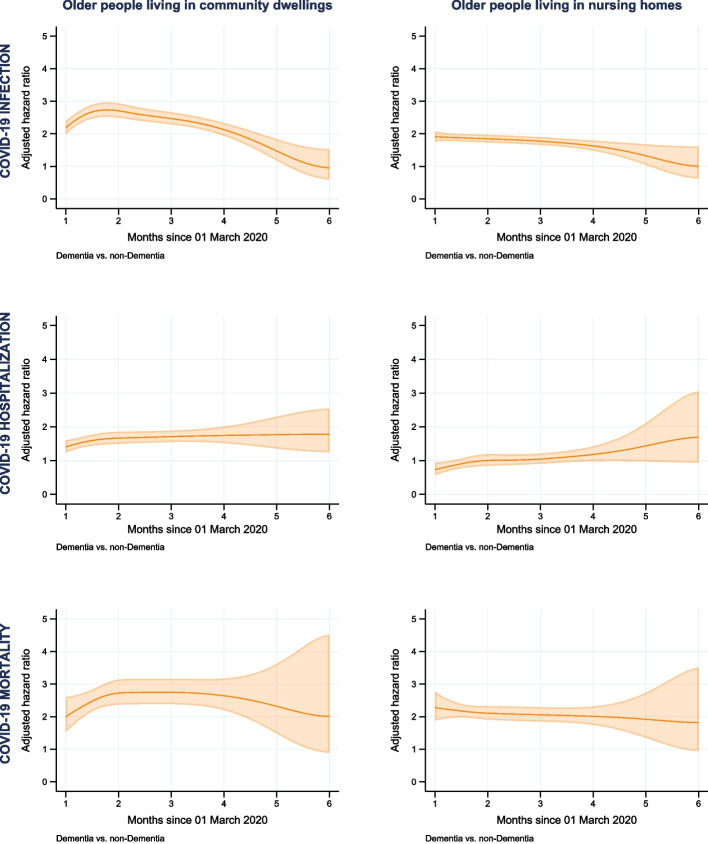
Adjusted hazard ratio of COVID-19 infection, hospitalization, and mortality between older persons with and without dementia (estimated with the flexible parametric competing-risks model)

Compared to people without dementia, people with dementia had significantly higher IRs of COVID-19 infection in both community dwellings (3.49 versus 0.97 per 1000 person-months) and nursing homes (13.19 versus 6.31 per 1000 person-months) (Fig. 1 and Table 2). The adjusted HRs of COVID-19 infection between people with and without dementia were displayed in Fig. 2 and Model 2, Supplementary eTable 6. In community dwellings, the adjusted HRs increased from 2.08 (95% CI 1.89, 2.29) in one month to 2.46 (95% CI 2.26, 2.68) in two months, and then decreased to 0.70 (95% CI 0.39, 1.26) in six months after the index date. In nursing homes, the adjusted HRs declined throughout the follow up period, from 1.89 (95% CI 1.74, 2.05) in one month to 0.91 (95% CI 0.56, 1.49) in six months after the index date.

**Table 2:** Numbers, time at risk and incidence rates of COVID-19 infection, hospitalization, and mortality

	Older people living in community dwellings			Older people living in nursing homes		
	*Number of events*	*Time at risk*	*Incidence rate (95% Confidence interval)*	*Number of events*	*Time at risk*	*Incidence rate (95% Confidence interval)*
COVID-19 infection						
*Non-Dementia*	11,676	12,014	0.97 (0.95, 0.99)	3252	515	6.31 (6.10, 6.53)
*Dementia*	812	232	3.49 (3.26, 3.75)	2430	184	13.19 (12.67, 13.73)
COVID-19 hospitalization						
*Non-Dementia*	9390	12,021	0.78 (0.77, 0.80)	919	525	1.75 (1.64, 1.87)
*Dementia*	573	233	2.46 (2.27, 2.67)	340	192	1.77 (1.60, 1.97)
COVID-19 mortality						
*Non-Dementia*	2491	12,049	0.21 (0.20, 0.22)	1334	523	2.55 (2.42, 2.69)
*Dementia*	281	234	1.20 (1.07, 1.35)	1176	189	6.23 (5.88, 6.60)

Time at risk was estimated in 1000 person-months

Incidence rate (95% Confidence interval) was presented as number of events per 1000 person-month

### COVID-19 hospitalization

In community dwellings, higher IRs of COVID-19 hospitalization was seen in people with dementia (2.46 per 1000 person-month) than in people without dementia (0.78 per 1000 person-month). Meanwhile, in nursing homes, the IRs of people with dementia was lower until two months after the index date, and then higher during the remaining follow-up period (Fig. 1 and Table 2). As can be seen in Fig. 2 and Model 2, Supplementary eTable 6, in community dwellings, people with dementia had significantly higher HRs of COVID-19 hospitalization, compared to people without dementia, increasing from 1.40 (95% CI 1.23, 1.59) in one month to 1.64 (95% CI 1.12, 2.41) in six months after the index date. In nursing homes, the HRs was lower in one month after the index date (HR 0.73, 95% CI 0.57–0.93), and higher than 1 in four months after the index date, but not statistically significant.

### COVID-19 mortality

Throughout the follow-up, people with dementia accounted for higher IRs of COVID-19 mortality compared to people without dementia in both community dwellings (1.20 versus 0.21 per 1000 person-month) and nursing homes (6.23 versus 2.55 per 1000 person-month) (Fig. 1 and Table 2). In community dwellings, the adjusted HRs showed people with dementia were at higher risk of COVID-19 mortality, which increasing from 1.96 (95% CI 1.49, 2.57) in one month to 2.39 (95% CI 2.05, 2.78) in two months and decreasing to 1.65 (95% CI 0.64, 4.26) in six months after the index date (Fig. 2 and Model 2, Supplementary eTable 6). In nursing homes, the downward trend in the adjusted HRs happened during the whole period, which declined from 2.27 (95% CI 1.87, 2.75) in one month to 1.69 (95% CI 0.85, 3.34) in six months after the index date.

### Sensitivity analysis

In the sensitivity analysis on the propensity score matched cohorts, similar trends occurred in both community dwellings and nursing homes (Model 3, Supplementary eTable 6). Among persons living in community dwellings, the adjusted HRs of COVID-19 infection or hospitalization increased and peaked in two months, then decreased in six months after the index date. The HRs of COVID-19 mortality increased gradually to 4.07 in six months after the index date. In nursing homes, the HRs of COVID-19 infection and mortality decreased between one and six months after the index date. In contrast, the HRs of COVID-19 hospitalization was higher than 1 from four months after the index date, however, not statistically significant.

## Discussion

In this nationwide study of persons aged 65 years old or above during the first wave of the COVID-19 pandemic in Sweden, dementia was associated with higher risks of COVID-19 infection and mortality, regardless of living arrangements. A higher risk of COVID-19 hospitalization was seen in persons with dementia living in community dwellings. In contrast, among persons living in nursing homes, dementia was not associated with COVID-19 hospitalization.

### COVID-19 infection

The higher risk of COVID-19 infection in persons with dementia might be explained by patient and health care system factors. First, it was difficult for older persons with dementia to follow recommendations from the public health agency to prevent the transmission of COVID-19, such as hand hygiene, social distancing, or self-isolation by staying at home [31]. Older persons with severe dementia were possibly unable to understand or remember the recommendations due to their cognitive impairment [31]. Behavioral and psychological symptoms of dementia, such as motor agitation, intrusiveness, or wandering, may constrain them from maintain self-isolation at home [31]. Furthermore, in the first wave of COVID-19 pandemic in Sweden, testing was mainly performed on persons with severe COVID-19 symptoms at hospitals [17]. Thus, being frail with dementia might increase the risk of ending up in hospitals and receiving testing.

From a health care system viewpoint, the higher risk of COVID-19 infection among older persons with dementia might be due to the failure to protect the more vulnerable groups in the COVID-19 strategy of Sweden [32, 33].

During the pandemic, Sweden never implemented a mandatory national lockdown, like other neighboring countries. Physical distancing was strongly recommended but only obligatory in restaurants and when visiting nursing homes. Older people were recommended to stay at home and follow the recommendations from the doctors when having COVID-19. If experiencing severe symptoms, they could contact emergency care and were hospitalized. The Swedish Corona Commission mentioned several failures of care for older persons in Sweden, such as the fragmented organization because of various actors with unclear responsibilities, or the lack of common information technology systems and communication channels [33]. Due to cognitive impairment and higher dependence in activities of daily living, older persons with dementia probably needed more support from caregivers providing home care services or at nursing homes. Thus, together with the shortage of protective equipment for these caregivers, the above failing probably made older persons with dementia exposed more to COVID-19 infection from caregivers providing home care services or at nursing homes. Our study was in line with a recent study in Swedish nursing homes, showing a 9% higher risk of COVID-19 infection in people with dementia [13]. Another study on persons aged 65 years and older in Lazio, Italy showed that dementia was associated with a 60% higher risk of COVID-19 infection [34].

### COVID-19 hospitalization

Regarding COVID-19 hospitalization, we observed higher risks among older persons with dementia living in community dwellings, but not among those living in nursing homes. Among community-dwelling persons, our findings were in concordance with a previous study on persons aged 18 years old or above in New York, US, which showed that persons with dementia had 3.6 times higher odds of having COVID-19 hospitalization, compared to persons without dementia [12]. Among nursing-home persons, another study on persons aged 65 years and older in US observed a 6% higher risk of COVID-19 hospitalization in persons with cognitive impairment, compared to those without cognitive impairment [35]. Our findings might be explained by the shortage of health care professionals and equipment at hospitals during the first wave of COVID-19 in Sweden. Both older persons with and without dementia having COVID-19 were recommended to stay at nursing homes, where they can get instant support from their caregivers. Then, scarce health care resources might be allocated to other diseases in hospitals. Additionally, COVID-19 infection was especially high in nursing homes during the first wave of COVID-19 in Sweden [13]. Thus, no matter whether older persons had dementia or not, they might be indicated to continue staying in nursing homes, to reduce the transmission to other vulnerable patients in hospitals. Alternatively, persons with dementia as well as without dementia might have received suboptimal care in nursing homes, which are their familiar living arrangement. This explanation is plausible as shown by the remarkable decrease of hospitalization among nursing home residents during the first wave of COVID-19 in Sweden [15]. This fact might imply that there was no difference in hospital admission between dementia and non-dementia when all other features, such as health status, dependency in activities of daily living or care settings, are equal.

### COVID-19 mortality

In terms of COVID-19 mortality, our findings concurred with various studies. Three meta-analyses on older persons with COVID-19 showed that dementia was associated with 3.75-, 2.96- and 3.69-times higher risk of COVID-19 mortality, respectively [5, 7, 36]. In Sweden, a recent study on older persons living in nursing homes found that dementia was associated with 25% higher risk of COVID-19 mortality [13]. Another Swedish study in geriatric care clinics in Stockholm, observed 68% higher COVID-19 mortality risk in older persons with dementia, compared to those without dementia [14]. Although the magnitude of estimates diverged, all studies stated that mechanisms for higher risk of COVID-19 mortality in older persons with dementia are vague and many-fold. First, severe COVID-19 outcomes were significantly associated with the “cytokine storm” of pronounced inflammation [37]. This might be exacerbated in persons with dementia, as neuroinflammation is a prominent feature of neurodegeneration [38]. Furthermore, the APOE4 allele, which is a main genetic risk factor for dementia [39], was also shown to be associated with four times increased risk of COVID-19 mortality [40]. The APOE4 allele also regulates the innate immune system and enhances pro-inflammatory responses, which relates to the “cytokine storm” of COVID-19 [39].

Another argument is that persons with dementia usually have other medical conditions, which are also risk factors for severe COVID-19 outcomes, including cerebrovascular diseases, diabetes, hypertension, heart failure, stroke [3, 41]. In our study, people with dementia had considerably higher percentages of these comorbidities, compared to people without dementia. Although we adjusted for these confounders and used propensity score matching in the sensitivity analysis, it was impossible to eliminate completely the influence of these characteristics on the outcomes. Additionally, the postponement in care for these comorbidities during the COVID-19 pandemic might also contribute to the higher vulnerability of people with dementia, compared to individuals without dementia with fewer comorbidities. For instance, inpatient episodes of cardiovascular, neurological, and respiratory diseases decreased by 9%, 8% and 31% in 2020, respectively [33]. Furthermore, persons with dementia were frailer, as shown by their significantly higher hospital frailty risk score, had a higher likelihood of obesity and physical inactivity [41]. Thus, they were probably more vulnerable and had worse immune system against the infection of COVID-19, resulting in more severe outcomes. The behavioral and psychological symptoms of dementia probably increased because of COVID-19 and isolation [42–44].

Moreover, neurological problems might restrain older persons with dementia from navigating care for COVID-19 or identifying COVID-19 early. They might need support from healthcare workers or their families to access care after COVID-19 infection. Not determining the COVID-19 diagnosis and accessing care punctually possibly resulted in the higher incidence rate of hospitalization and death among people with dementia. This explanation is plausible because we observed higher HRs of COVID-19 hospitalization and mortality between people with and without dementia in community-dwelling persons, compared to nursing-homes individuals. In nursing homes, instant support from caregivers might decrease the difference in the risk of severe COVID-19 outcomes between these two groups.

### Strengths and limitations

The linkage of nationwide registries is the main strength of this study. It enabled a large sample size and removed non-participation bias. In contrast to self-reported data, there was no recall bias. Extended follow-up and details of drug prescription and diagnosis are other additional strengths.

Weaknesses of this study were that the severity of dementia was not considered because these national registries only report the diagnosis or drug dispensing. However, we tried to mitigate bias by adjusting for comorbidities and hospital frailty risk score. Second, many persons having COVID-19 were not recorded in national registries because they might have COVID-19 without any severe syndrome or might not be identified due to the deficiency of healthcare resources in the first wave. We have attempted to handle this issue by finding COVID-19 cases from different registers to broaden our detection of persons having COVID-19. Furthermore, the absence of primary care data may have resulted in an underestimation of the actual incidence and severity of outcomes.

## Conclusions

In the first wave of COVID-19 in Sweden, older persons with dementia had a higher risk of COVID-19 infection and mortality compared to the general older population, regardless of living arrangements. Dementia was associated with a higher risk of COVID-19 hospitalization among community-dwelling persons, but not among those living in nursing homes. Future studies on the provision of care services for older persons with dementia are important to improve the quality of care, as well as to enhance the preparedness for other pandemics.

## Supplementary Information

Supplementary Material 1.

## Data Availability

The data that support the findings of this study are not publicly available because the Swedish law do not allow for sharing the data from the Swedish national registries.
