# Supplementary material for: Impact of dementia on risks of COVID-19 infection and outcomes among older adults in Sweden
Source: BMC Geriatr. 2025 Nov 4;25:843. doi: 10.1186/s12877-025-06576-3 (PMC12584336; doi:10.1186/s12877-025-06576-3)
Supplement: Supplementary file 1 — Supplementary Material 1. [file 12877_2025_6576_MOESM1_ESM.docx]

**Impact of dementia on risks of COVID-19 infection and outcomes among older adults in Sweden**

**SUPPLEMENTARY MATERIALS**

[eTable 1. Data source of the study 3](#_Toc199546545)

[eTable 2. International Classification of Diseases (ICD) 10 and Anatomical Therapeutic Chemical (ATC) codes for dementia, COVID-19 and comorbidities (10-12) 4](#_Toc199546546)

[eTable 3. Diagnosis codes and weights used to calculate the Hospital Frailty Risk Score (13) 5](#_Toc199546547)

[eTable 4. The RECORD statement – checklist of items, extended from the STROBE statement, that should be reported in observational studies using routinely collected health data (1) 7](#_Toc199546548)

[eTable 5. Characteristics of the propensity score matched cohort 9](#_Toc199546549)

[eTable 6. Hazard Ratios of COVID-19 infection, hospitalization, and mortality between older people with and without dementia 10](#_Toc199546550)

[eFigure 1. The study design diagram 11](#_Toc199546551)

[eFigure 2. The selection of participants 12](#_Toc199546552)

[eFigure 3. The standardized mean differences before and after propensity score matching 13](#_Toc199546553)

[eFigure 4. Number of older people who had COVID-19 infection, hospitalization and mortality in community dwellings and nursing homes 14](#_Toc199546554)

[References for Supplements 15](#_Toc199546555)

## eTable 1. Data source of the study

| **Register** | **Founded year** | **Characteristics** | **Externally validated** | **Coverage** | **Data extracted for our study** |
| --- | --- | --- | --- | --- | --- |
| The Total Population Register (2) | 1968 | The register provides complete population-based data, which consists of 100 % of births and deaths, 95 % of immigrations and 91 % of emigrations  Data are reported within 30 days and with a higher proportion over time. | Yes | Nationwide | Sex  Month and year of birth  Country of birth  Municipality of residence  Marital status  Date of immigration and emigration |
| The Swedish Longitudinal Integrated Database for Health Insurance and Labor Market Studies (LISA) (3) | 1990 | LISA is updated annually with a roughly 15-month delay.  LISA contains data of all individuals aged ≥ 16 years who are registered in Sweden as of December 31 each year regarding education, income, occupation and so on. | Yes | Nationwide | Highest educational attainment  Disposable individual income |
| The National Patient Register (4) | 1964 | The register collects data from 99% of all somatic and psychiatric hospital discharges, and over 80% of hospital-based outpatient care. | About 85-95% of all diagnoses are valid | Nationwide | Date of admission and discharge  Main diagnosis  Secondary diagnoses |
| The National Prescribed Drug Register (5, 6) | 2005 | The register reports contains all dispensed prescription drugs in pharmacy in Sweden (equal to 85% of all sold defined daily doses)  The register does not cover the remaining 15%, including over-the-counter drugs and drugs administered in hospitals. | Yes | Nationwide | Date of prescription  Date of dispensing  ATC code |
| The National Cause of Death Register (7) | 1952 | The register reports all deaths, with an underlying cause of death. | Yes | Nationwide | Cause of death  Date of death |
| The Swedish Social Services Register (8) | 2007 | This register reports all social services provided to older people or people with disabilities.  Data are reported monthly by the municipalities in Sweden. | Yes | Nationwide | Living arrangement (ordinary homes vs. nursing homes)  Month and year of report |
| The Swedish Registry for Infectious Diseases - SmiNet (9) | 1997 | The register manages about 60 infectious diseases that are notifiable according to the Infection Control Act in Sweden.  Data are reported monthly by doctors and laboratories in Sweden. | Yes | Nationwide | Clinically confirmed COVID-19 infection  Date of COVID-19 test, diagnosis and statistics |

## eTable 2. International Classification of Diseases (ICD) 10 and Anatomical Therapeutic Chemical (ATC) codes for dementia, COVID-19 and comorbidities (10-12)

|  | **ICD-10 codes from the National Patient Register** | **ATC codes from the National Prescribed Drug Register** |
| --- | --- | --- |
| **Asthma** | J45 | R03DC R03BC |
| **Atrial fibrillation** | I48 |  |
| **Cerebrovascular diseases** | G45 G46  I60-I64 I67 I69 |  |
| **Chronic infectious diseases** | A15-A19 A30 A31 A50 A52 A53 A65-A67 A692 A81  B20-B24 B381 B391 B401 B572-B575 B65 B92 B94  J65  M863-M866 | J04A (excluded J04AB01 J04AB02 J04AB03 J04AC) |
| **Chronic kidney diseases** | I120 I130 I131 I132 I139  N01 N03 N04 N05 N07 N08 N11 N183 N184 N185 N189  Q60 Q611-Q615 Q618 Q619  Z905 Z940 |  |
| **Chronic liver diseases** | B18  K70 K713 K714 K715 K717 K721 K73 K74 K753 K754 K758 K761 K766 K767 K778  Q446  Z944 |  |
| **COPD, emphysema, chronic bronchitis** | J41-J44 J47 | R03BB |
| **COVID-19** | U071 U072 |  |
| **Dementia** | F00-F03 F051  G30 G31 | N06DA N06DX01 |
| **Depression and mood diseases** | F30-F34 F38 F39 F412 |  |
| **Diabetes** | E10 E11 E13 E14 E891 | A10 |
| **Heart failure** | I110 I130 I132 I27 I280 I42 I43 I50 I515 I517 I528 Z941 Z943 |  |
| **Hypertension** | I10-I13 I15 |  |
| **Ischemic heart diseases** | I20 I21 I22 I24 I25 Z951 Z955 | C01DA C01EB18 |
| **Obesity** | E66 |  |
| **Osteoporosis** | M80-M82 | M05BA M05BB M05BX03 M05BX53 |
| **Parkinson and parkinsonism** | G20-G23 | N04BA N04BX |
| **Peripheral vascular diseases** | I702 I73 I792 I798 I731 I738 | B01AC23 |

## eTable 3. Diagnosis codes and weights used to calculate the Hospital Frailty Risk Score (13)

| **Diagnosis** | **ICD10-codes** | **Weight** |
| --- | --- | --- |
| Abnormal results of function studies | R94 | 1.4 |
| Abnormalities of gait and mobility | R26 | 2.6 |
| Abnormalities of heart beat | R00 | 0.7 |
| Acute renal failure | N17 | 1.8 |
| Agent resistant to penicillin and related antibiotics | U80 | 0.8 |
| Artificial opening status | Z93 | 1.0 |
| Blindness and low vision | H54 | 1.9 |
| Calculus of kidney and ureter | N20 | 0.7 |
| Care involving use of rehabilitation procedures | Z50 | 2.1 |
| Carrier of infectious disease | Z22 | 1.7 |
| Cellulitis | L03 | 2.0 |
| Cerebral Infarction | I63 | 0.8 |
| Chronic renal failure | N18 | 1.4 |
| Complications of genitourinary prosthetic devices, implants and grafts | T83 | 2.4 |
| Convulsions, not elsewhere classified | R56 | 2.6 |
| Decubitus ulcer | L89 | 1.7 |
| Deficiency of other B group vitamins | E53 | 1.9 |
| Dependence on enabling machines and devices | Z99 | 0.8 |
| Depressive episode | F32 | 0.5 |
| Diarrhoea and gastroenteritis of presumed infectious origin | A09 | 1.1 |
| Disorders of mineral metabolism | E83 | 0.4 |
| Duodenal ulcer | K26 | 1.6 |
| Dysphagia | R13 | 0.8 |
| Epilepsy | G40 | 1.5 |
| Exposure to unspecified factor | X59 | 1.5 |
| Fall involving bed | W06 | 1.1 |
| Fall on and from stairs and steps | W10 | 0.9 |
| Fall on same level from slipping, tripping and stumbling | W01 | 0.9 |
| Fever of unknown origin | R50 | 0.1 |
| Fracture of femur | S72 | 1.4 |
| Fracture of lumbar spine and pelvis | S32 | 1.4 |
| Fracture of rib(s), sternum and thoracic spine | S22 | 1.8 |
| Fracture of shoulder and upper arm | S42 | 2.3 |
| Gangrene, not elsewhere classified | R02 | 1.0 |
| Hemiplegia | G81 | 4.4 |
| Hypotension | I95 | 1.6 |
| Intracranial injury | S06 | 2.4 |
| Mental and behavioural disorders due to use of alcohol | F10 | 0.7 |
| Nausea and vomiting | R11 | 0.3 |
| Nosocomial condition | Y95 | 1.2 |
| Open wound of forearm | S51 | 0.5 |
| Open wound of head | S01 | 1.1 |
| Osteoporosis with pathological fracture | M80 | 0.8 |
| Osteoporosis without pathological fracture | M81 | 1.4 |
| Other abnormal findings of blood chemistry | R79 | 0.6 |
| Other anaemias | D64 | 0.4 |
| Other and unspecified injuries of head | S09 | 1.2 |
| Other arthrosis | M19 | 1.5 |
| Other bacterial agents as the cause of diseases classified to other chapters (secondary code) | B96 | 2.9 |
| Other bacterial intestinal infections | A04 | 1.1 |
| Other cerebrovascular diseases | I67 | 2.6 |
| Other diseases of digestive system | K92 | 0.8 |
| Other disorders of fluid, electrolyte and acid-base balance | E87 | 2.3 |
| Other disorders of kidney and ureter, not elsewhere classified | N28 | 1.3 |
| Other disorders of pancreatic internal secretion | E16 | 1.4 |
| Other disorders of urinary system (includes urinary tract infection and urinary incontinence) | N39 | 3.2 |
| Other fall on same level | W18 | 2.1 |
| Other functional intestinal disorders | K59 | 1.8 |
| Other hearing loss | H91 | 0.9 |
| Other joint disorders, not elsewhere classified | M25 | 2.3 |
| Other local infections of skin and subcutaneous tissue | L08 | 0.4 |
| Other medical procedures as the cause of abnormal reaction | Y84 | 0.7 |
| Other noninfective gastroenteritis and colitis | K52 | 0.3 |
| Other septicaemia | A41 | 1.6 |
| Other soft tissue disorders, not elsewhere classified | M79 | 1.1 |
| Other symptoms and signs involving cognitive functions and awareness | R41 | 2.7 |
| Other symptoms and signs involving general sensations and perceptions | R44 | 1.6 |
| Other symptoms and signs involving the nervous and musculoskeletal systems (R29·6 Tendency to fall) | R29 | 3.6 |
| Parkinson's disease | G20 | 1.8 |
| Personal history of other diseases and conditions | Z87 | 1.5 |
| Personal history of risk-factors, not elsewhere classified | Z91 | 0.5 |
| Pneumonia, organism unspecified | J18 | 1.1 |
| Pneumonitis due to solids and liquids | J69 | 1.0 |
| Polyarthrosis | M15 | 0.4 |
| Problems related to care-provider dependency | Z74 | 1.1 |
| Problems related to life-management difficulty | Z73 | 0.6 |
| Problems related to medical facilities and other health care | Z75 | 2.0 |
| Problems related to social environment | Z60 | 1.8 |
| Respiratory failure, not elsewhere classified | J96 | 1.5 |
| Retention of urine | R33 | 1.3 |
| Scoliosis | M41 | 0.9 |
| Senility | R54 | 2.2 |
| Sequelae of cerebrovascular disease (secondary codes) | I69 | 3.7 |
| Somnolence, stupor and coma | R40 | 2.5 |
| Speech disturbances, not elsewhere classified | R47 | 1.0 |
| Spinal stenosis (secondary code only) | M48 | 0.5 |
| Streptococcus and staphylococcus as the cause of diseases classified to other chapters | B95 | 1.7 |
| Superficial injury of head | S00 | 3.2 |
| Superficial injury of lower leg | S80 | 2.0 |
| Symptoms and signs concerning food and fluid intake | R63 | 0.9 |
| Symptoms and signs involving emotional state | R45 | 1.2 |
| Syncope and collapse | R55 | 1.8 |
| Thyrotoxicosis (hyperthyroidism) | E05 | 0.9 |
| Transient cerebral ischaemic attacks and related syndromes | G45 | 1.2 |
| Ulcer of lower limb, not elsewhere classified | L97 | 1.6 |
| Unknown and unspecified causes of morbidity | R69 | 1.3 |
| Unspecified acute lower respiratory infection | J22 | 0.7 |
| Unspecified fall | W19 | 3.2 |
| Unspecified haematuria | R31 | 3.0 |
| Unspecified renal failure | N19 | 1.6 |
| Unspecified urinary incontinence | R32 | 1.2 |
| Vitamin D deficiency | E55 | 1.0 |
| Volume depletion | E86 | 2.3 |

## eTable 4. The RECORD statement – checklist of items, extended from the STROBE statement, that should be reported in observational studies using routinely collected health data (1)

| **RECORD items** | | **Details** | **Location** |
| --- | --- | --- | --- |
| **Title and abstract** | 1 | (1)The type of data used should be specified in the title or abstract. When possible, the name of the databases used should be included. | Abstract |
|  |  | (2) If applicable, the geographic region and timeframe within which the study took place should be reported in the title or abstract. | Abstract |
|  |  | (3) If linkage between databases was conducted for the study, this should be clearly stated in the title or abstract. | Abstract |
| **Introduction** | | |  |
| Background/rationale | 2 | Explain the scientific background and rationale for the investigation being reported | Introduction |
| Objectives | 3 | State specific objectives, including any prespecified hypotheses | Introduction > Last paragraph |
| **Methods** | | |  |
| Study design | 4 | Present key elements of study design early in the paper | Methods > Study design and data sources |
| Setting | 5 | Describe the setting, locations, and relevant dates, including periods of recruitment, exposure, follow-up, and data collection | Methods > Study design and data sources |
| Participants | 6 | (*1*) The methods of study population selection (such as codes or algorithms used to identify subjects) should be listed in detail. If this is not possible, an explanation should be provided. | Methods > Participants |
|  |  | (*2*) Any validation studies of the codes or algorithms used to select the population should be referenced. If validation was conducted for this study and not published elsewhere, detailed methods and results should be provided. | Not applicable |
|  |  | (3) If the study involved linkage of databases, consider use of a flow diagram or other graphical display to demonstrate the data linkage process, including the number of individuals with linked data at each stage. | Figure 1 |
| Variables | 7 | A complete list of codes and algorithms used to classify exposures, outcomes, confounders, and effect modifiers should be provided. If these cannot be reported, an explanation should be provided. | Methods > Exposure & Covariates, Outcomes |
| Data sources/ measurement | 8* | For each variable of interest, give sources of data and details of methods of assessment (measurement). Describe comparability of assessment methods if there is more than one group | Methods > Study design and data sources & eTable 2 |
| Bias | 9 | Describe any efforts to address potential sources of bias | Discussion > Strength & Limitations |
| Study size | 10 | Explain how the study size was arrived at | Methods > Participants & Figure 1 |
| Quantitative variables | 11 | Explain how quantitative variables were handled in the analyses. If applicable, describe which groupings were chosen and why | Methods > Exposure & Covariates, Outcomes |
| Statistical methods | 12 | (*a*) Describe all statistical methods, including those used to control for confounding | Methods > Statistical analysis |
|  |  | (*b*) Describe any methods used to examine subgroups and interactions | Methods > Statistical analysis |
|  |  | (*c*) Explain how missing data were addressed | Not applicable |
|  |  | (*d*) If applicable, explain how loss to follow-up was addressed | Not applicable |
|  |  | (*e*) Describe any sensitivity analyses | Methods > Statistical analysis |
| Data access and cleaning methods |  | Authors should describe the extent to which the investigators had access to the database population used to create the study population. | Methods > Study design and data sources & eTable 2 |
|  |  | Authors should provide information on the data cleaning methods used in the study. | Not applicable |
|  |  | State whether the study included person-level, institutional-level, or other data linkage across two or more databases. The methods of linkage and methods of linkage quality evaluation should be provided. | Methods > Study design and data sources & eTable 2 |
| **Results** | | |  |
| Participants | 13* | (a) Describe in detail the selection of the persons included in the study (i.e., study population selection) including filtering based on data quality, data availability and linkage. The selection of included persons can be described in the text and/or by means of the study flow diagram. | Methods > Participants & Figure 1 |
|  |  | (b) Give reasons for non-participation at each stage | Methods > Participants & Figure 1 |
|  |  | (c) Consider use of a flow diagram | Methods > Participants & Figure 1 |
| Descriptive data | 14* | (a) Give characteristics of study participants (eg demographic, clinical, social) and information on exposures and potential confounders | Results > Characteristics of selected participants & Table 1 |
|  |  | (b) Indicate number of participants with missing data for each variable of interest | Not applicable |
|  |  | (c) Summarise follow-up time (eg, average and total amount) | Table 2 |
| Outcome data | 15* | Report numbers of outcome events or summary measures over time | Table 2 |
| Main results | 16 | (*a*) Give unadjusted estimates and, if applicable, confounder-adjusted estimates and their precision (eg, 95% confidence interval). Make clear which confounders were adjusted for and why they were included | Table 2 |
|  |  | (*b*) Report category boundaries when continuous variables were categorized | Not applicable |
|  |  | (*c*) If relevant, consider translating estimates of relative risk into absolute risk for a meaningful time period | Not applicable |
| Other analyses | 17 | Report other analyses done—eg analyses of subgroups and interactions, and sensitivity analyses | Results > Sensitivity analyses |
| **Discussion** | | |  |
| Key results | 18 | Summarise key results with reference to study objectives | Discussion > First paragraph |
| Limitations | 19 | Discuss the implications of using data that were not created or collected to answer the specific research question(s). Include discussion of misclassification bias, unmeasured confounding, missing data, and changing eligibility over time, as they pertain to the study being reported. | Discussion > Strength & Limitations |
| Interpretation | 20 | Give a cautious overall interpretation of results considering objectives, limitations, multiplicity of analyses, results from similar studies, and other relevant evidence | Discussion |
| Generalisability | 21 | Discuss the generalisability (external validity) of the study results | Discussion |
| **Other information** | | |  |
| Funding | 22 | Give the source of funding and the role of the funders for the present study and, if applicable, for the original study on which the present article is based | Acknowledgements: Funding |
| Accessibility of protocol, raw data, and programming code |  | Authors should provide information on how to access any supplemental information such as the study protocol, raw data, or programming code. | Acknowledgements: Availability of data and materials |

## eTable 5. Characteristics of the propensity score matched cohort

|  | **Older people living in community dwellings** | | **Older people living in nursing homes** | |
| --- | --- | --- | --- | --- |
|  | *non-Dementia* | *Dementia* | *non-Dementia* | *Dementia* |
| N | 40759 | 40759 | 31143 | 31143 |
| Age, years, mean ± SD | 81.3 ± 6.8 | 81.2 ± 6.8 | 85.3 ± 7.3 | 85.1 ± 7.3 |
| Female, n (%) | 22569 (55.4) | 22169 (54.4) | 20725 (66.5) | 20431 (65.6) |
| Living areas, n (%) |  |  |  |  |
| Urban | 12402 (30.4) | 12512 (30.7) | 8749 (28.1) | 9051 (29.1) |
| Intermediate | 15906 (39.0) | 15779 (38.7) | 12961 (41.6) | 12699 (40.8) |
| Rural | 12451 (30.5) | 12468 (30.6) | 9433 (30.3) | 9393 (30.2) |
| Cohabitation status, n (%) |  |  |  |  |
| Cohabiting | 25010 (61.4) | 25432 (62.4) | 9161 (29.4) | 9857 (31.7) |
| Living alone | 15749 (38.6) | 15327 (37.6) | 21982 (70.6) | 21286 (68.3) |
| Education, n (%) |  |  |  |  |
| University/College | 9248 (22.7) | 9190 (22.5) | 5181 (16.6) | 5425 (17.4) |
| Secondary education | 15972 (39.2) | 15967 (39.2) | 11297 (36.3) | 11281 (36.2) |
| Compulsory education | 14993 (36.8) | 14917 (36.6) | 14210 (45.6) | 13936 (44.7) |
| Unknown | 546 (1.3) | 685 (1.7) | 455 (1.5) | 501 (1.6) |
| Hospital Frailty Risk Score, n (%) |  |  |  |  |
| Low | 17791 (43.6) | 17614 (43.2) | 7996 (25.7) | 8056 (25.9) |
| Moderate | 18281 (44.9) | 17939 (44.0) | 15758 (50.6) | 15524 (49.8) |
| High | 4687 (11.5) | 5206 (12.8) | 7389 (23.7) | 7563 (24.3) |
| Number of drugs, n (%) |  |  |  |  |
| <5 | 16948 (41.6) | 16781 (41.2) | 6604 (21.2) | 6741 (21.6) |
| 5-9 | 19720 (48.4) | 19436 (47.7) | 18189 (58.4) | 18122 (58.2) |
| ≥10 | 4091 (10.0) | 4542 (11.1) | 6350 (20.4) | 6280 (20.2) |
| Chronic comorbidities, n (%) |  |  |  |  |
| Asthma | 1866 (4.6) | 2103 (5.2) | 1678 (5.4) | 1739 (5.6) |
| Atrial fibrillation | 7879 (19.3) | 8130 (19.9) | 7201 (23.1) | 7237 (23.2) |
| Cerebrovascular diseases | 8444 (20.7) | 8594 (21.1) | 8573 (27.5) | 8529 (27.4) |
| Chronic infectious diseases | 412 (1.0) | 478 (1.2) | 354 (1.1) | 352 (1.1) |
| Chronic kidney diseases | 1792 (4.4) | 1987 (4.9) | 1716 (5.5) | 1688 (5.4) |
| Chronic liver diseases | 290 (0.7) | 343 (0.8) | 242 (0.8) | 229 (0.7) |
| COPD | 2503 (6.1) | 2759 (6.8) | 2304 (7.4) | 2278 (7.3) |
| Depression | 4103 (10.1) | 4130 (10.1) | 4153 (13.3) | 4068 (13.1) |
| Diabetes | 6851 (16.8) | 7068 (17.3) | 5598 (18.0) | 5722 (18.4) |
| Heart failure | 4589 (11.3) | 4947 (12.1) | 4791 (15.4) | 4805 (15.4) |
| Hypertension | 21933 (53.8) | 21906 (53.7) | 18824 (60.4) | 18716 (60.1) |
| Ischemic heart diseases | 7970 (19.6) | 8338 (20.5) | 6551 (21.0) | 6673 (21.4) |
| Obesity | 843 (2.1) | 1000 (2.5) | 637 (2.0) | 640 (2.1) |
| Osteoporosis | 2718 (6.7) | 2900 (7.1) | 3270 (10.5) | 3384 (10.9) |
| Parkinson and parkinsonism | 2125 (5.2) | 2303 (5.7) | 1564 (5.0) | 1604 (5.2) |
| Peripheral vascular diseases | 1184 (2.9) | 1337 (3.3) | 1161 (3.7) | 1166 (3.7) |
| SD – Standard deviation  COPD - Chronic obstructive pulmonary disease | | | | |

## eTable 6. Hazard Ratios of COVID-19 infection, hospitalization, and mortality between older people with and without dementia

|  | **COVID-19 INFECTION** | | **COVID-19 HOSPITALIZATION** | | **COVID-19 MORTALITY** | |
| --- | --- | --- | --- | --- | --- | --- |
| **Months since**  **01 March 2020** | **Older people living in community dwellings** | **Older people living in**  **nursing homes** | **Older people living in community dwellings** | **Older people living in nursing homes** | **Older people living in community dwellings** | **Older people living in nursing homes** |
| **Model 1** |  |  |  |  |  |  |
| 1 month | 3.60 (3.28, 3.96) | 2.23 (2.05, 2.41) | 2.82 (2.48, 3.20) | 0.78 (0.61, 1.00) | 5.02 (3.83, 6.58) | 2.68 (2.22, 3.25) |
| 2 months | 4.24 (3.90, 4.61) | 2.11 (1.98, 2.24) | 3.25 (2.92, 3.61) | 1.04 (0.88, 1.23) | 6.08 (5.23, 7.05) | 2.41 (2.19, 2.65) |
| 3 months | 3.83 (3.54, 4.14) | 2.01 (1.88, 2.15) | 3.30 (2.98, 3.65) | 1.07 (0.93, 1.23) | 6.04 (5.19, 7.03) | 2.34 (2.10, 2.60) |
| 4 months | 3.22 (2.92, 3.54) | 1.84 (1.68, 2.02) | 3.32 (2.87, 3.84) | 1.18 (0.99, 1.41) | 5.75 (4.72, 7.01) | 2.27 (1.98, 2.61) |
| 5 months | 2.08 (1.63, 2.66) | 1.48 (1.17, 1.87) | 3.30 (2.49, 4.36) | 1.40 (0.95, 2.05) | 4.98 (3.04, 8.15) | 2.15 (1.51, 3.05) |
| 6 months | 1.20 (0.67, 2.14) | 1.08 (0.66, 1.76) | 3.28 (2.24, 4.81) | 1.62 (0.89, 2.96) | 4.19 (1.62, 10.85) | 2.00 (1.01, 3.95) |
| **Model 2** |  |  |  |  |  |  |
| 1 month | 2.08 (1.89, 2.29) | 1.89 (1.74, 2.05) | 1.40 (1.23, 1.59) | 0.73 (0.57, 0.93) | 1.96 (1.49, 2.57) | 2.27 (1.87, 2.75) |
| 2 months | 2.46 (2.26, 2.68) | 1.79 (1.68, 1.91) | 1.62 (1.45, 1.80) | 0.96 (0.81, 1.14) | 2.39 (2.05, 2.78) | 2.05 (1.86, 2.25) |
| 3 months | 2.22 (2.05, 2.41) | 1.71 (1.60, 1.83) | 1.65 (1.49, 1.82) | 0.99 (0.86, 1.15) | 2.38 (2.04, 2.78) | 1.99 (1.78, 2.21) |
| 4 months | 1.87 (1.69, 2.06) | 1.57 (1.43, 1.72) | 1.66 (1.43, 1.92) | 1.10 (0.92, 1.32) | 2.26 (1.85, 2.77) | 1.93 (1.68, 2.21) |
| 5 months | 1.21 (0.95, 1.55) | 1.26 (0.99, 1.59) | 1.65 (1.25, 2.19) | 1.30 (0.88, 1.91) | 1.96 (1.19, 3.21) | 1.82 (1.28, 2.58) |
| 6 months | 0.70 (0.39, 1.26) | 0.91 (0.56, 1.49) | 1.64 (1.12, 2.41) | 1.50 (0.82, 2.74) | 1.65 (0.64, 4.26) | 1.69 (0.85, 3.34) |
| **Model 3** |  |  |  |  |  |  |
| 1 month | 1.97 (1.68, 2.30) | 1.93 (1.74, 2.15) | 1.47 (1.20, 1.80) | 0.79 (0.59, 1.04) | 2.08 (1.59, 2.72) | 2.10 (1.65, 2.68) |
| 2 months | 2.39 (2.07, 2.76) | 1.76 (1.62, 1.90) | 1.71 (1.44, 2.04) | 0.98 (0.81, 1.20) | 2.21 (1.78, 2.75) | 2.00 (1.76, 2.26) |
| 3 months | 2.28 (1.99, 2.61) | 1.64 (1.51, 1.78) | 1.71 (1.46, 2.01) | 0.99 (0.84, 1.17) | 2.41 (1.91, 3.03) | 1.93 (1.69, 2.20) |
| 4 months | 2.03 (1.67, 2.46) | 1.45 (1.29, 1.64) | 1.67 (1.32, 2.10) | 1.05 (0.86, 1.28) | 2.76 (1.86, 4.10) | 1.80 (1.51, 2.14) |
| 5 months | 1.57 (1.05, 2.36) | 1.10 (0.83, 1.46) | 1.60 (1.07, 2.39) | 1.15 (0.73, 1.82) | 3.49 (1.59, 7.66) | 1.55 (1.02, 2.37) |
| 6 months | 1.26 (0.68, 2.34) | 0.77 (0.45, 1.32) | 1.56 (0.95, 2.57) | 1.28 (0.58, 2.79) | 4.07 (1.39, 11.92) | 1.30 (0.60, 2.80) |
| Data were presented as hazard ratio (95% confidence interval).  Model 1. Unadjusted flexible parametric competing-risks model.  Model 2. Flexible parametric competing-risks model adjusted for age at the start of the first COVID-19 pandemic, sex, living areas, cohabitation status, education, hospital frailty risk score, number of drugs and comorbidities.  Model 3. Unadjusted flexible parametric competing-risks model (propensity score matched cohort) | | | | | | |

## eFigure 1. The study design diagram


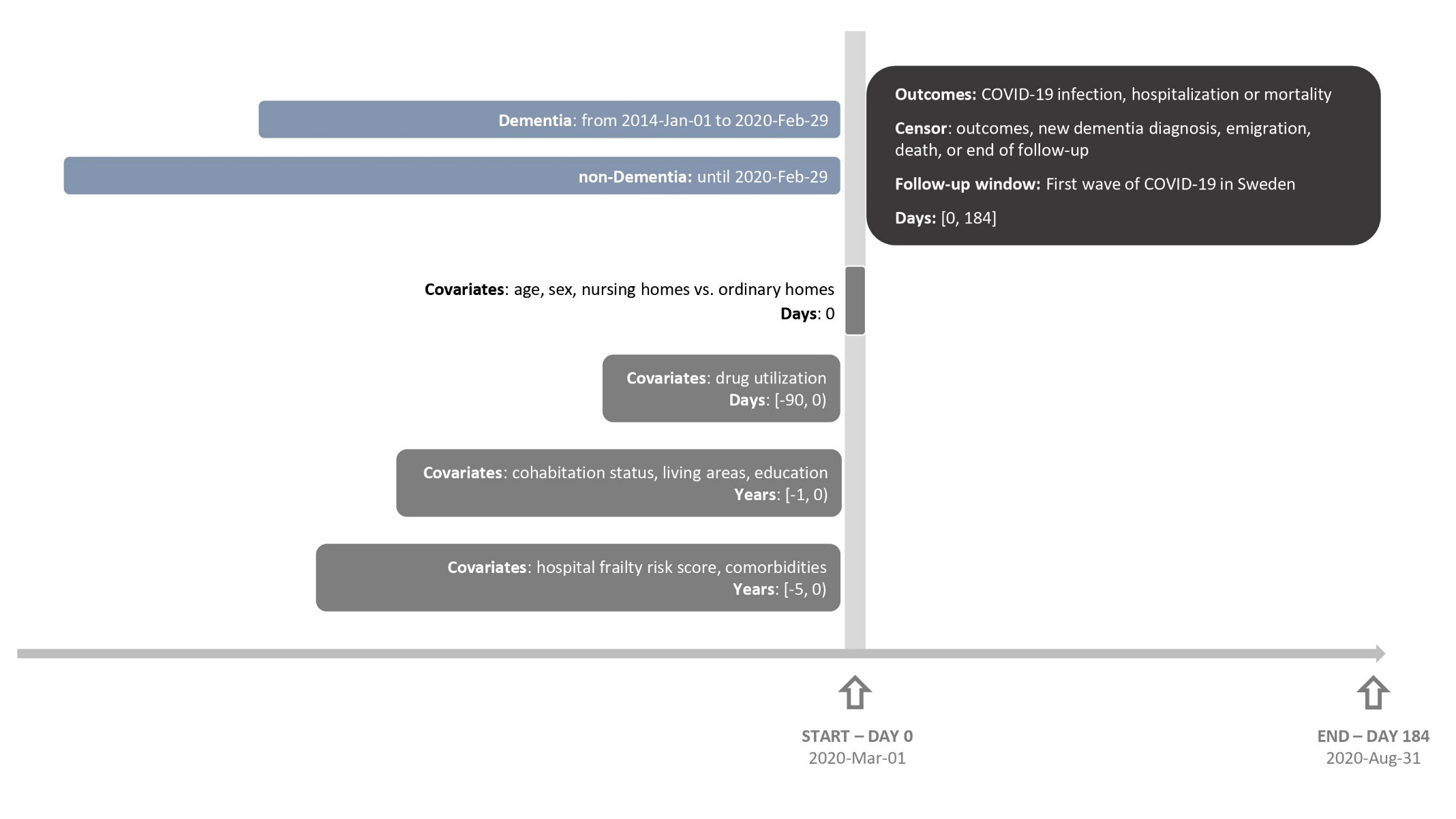


## eFigure 2. The selection of participants


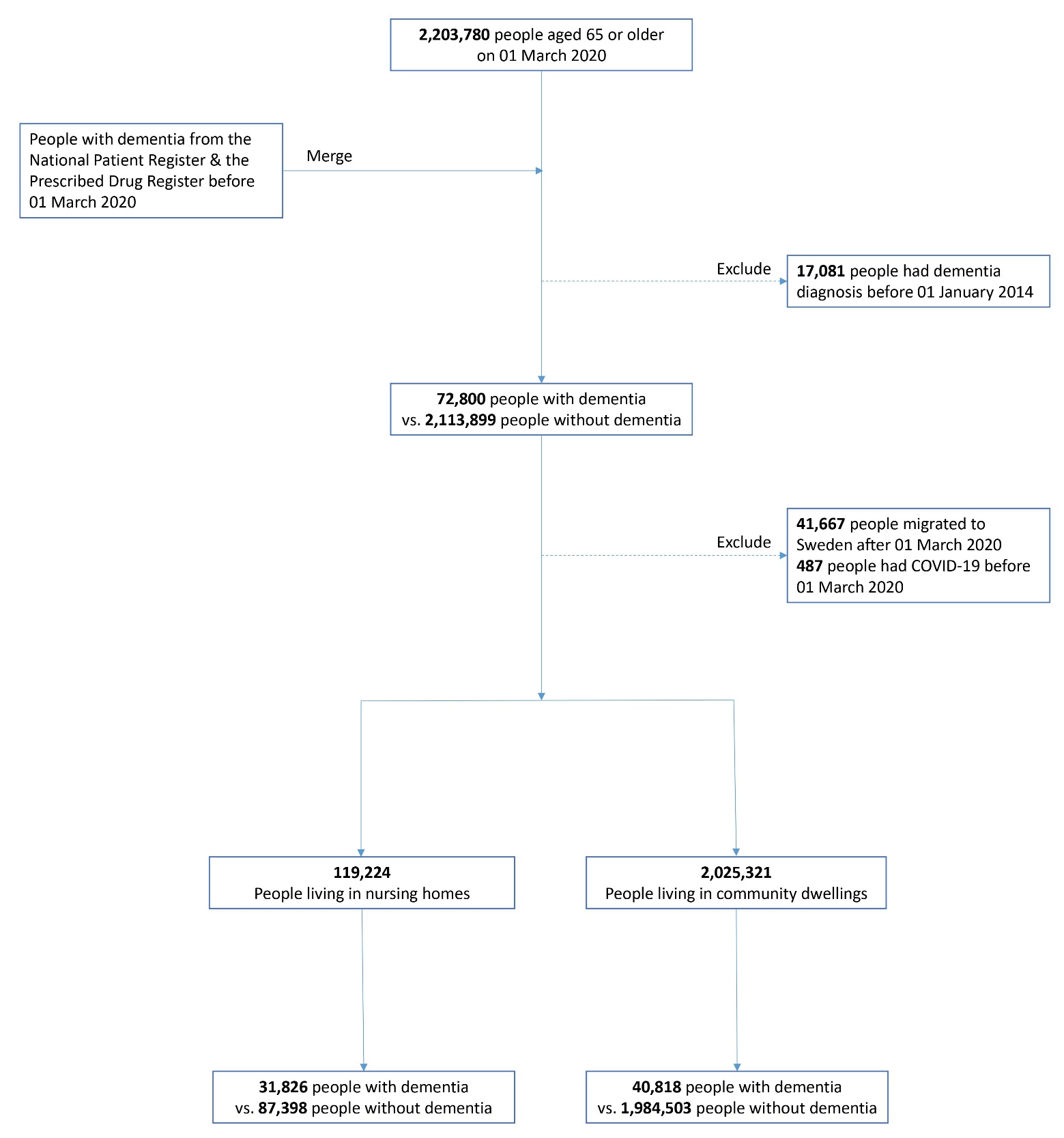


## eFigure 3. The standardized mean differences before and after propensity score matching


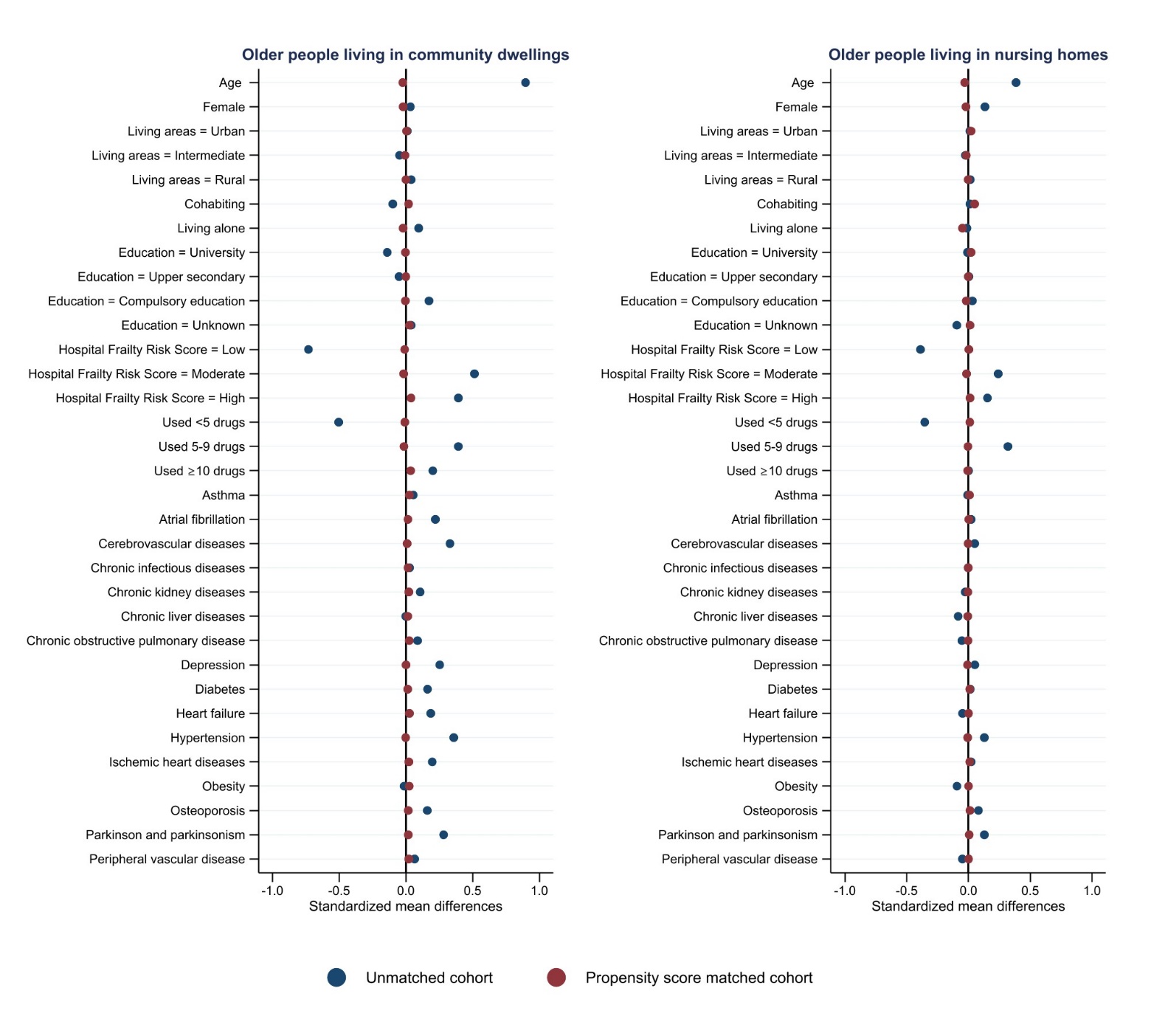


## eFigure 4. Number of older people who had COVID-19 infection, hospitalization and mortality in community dwellings and nursing homes


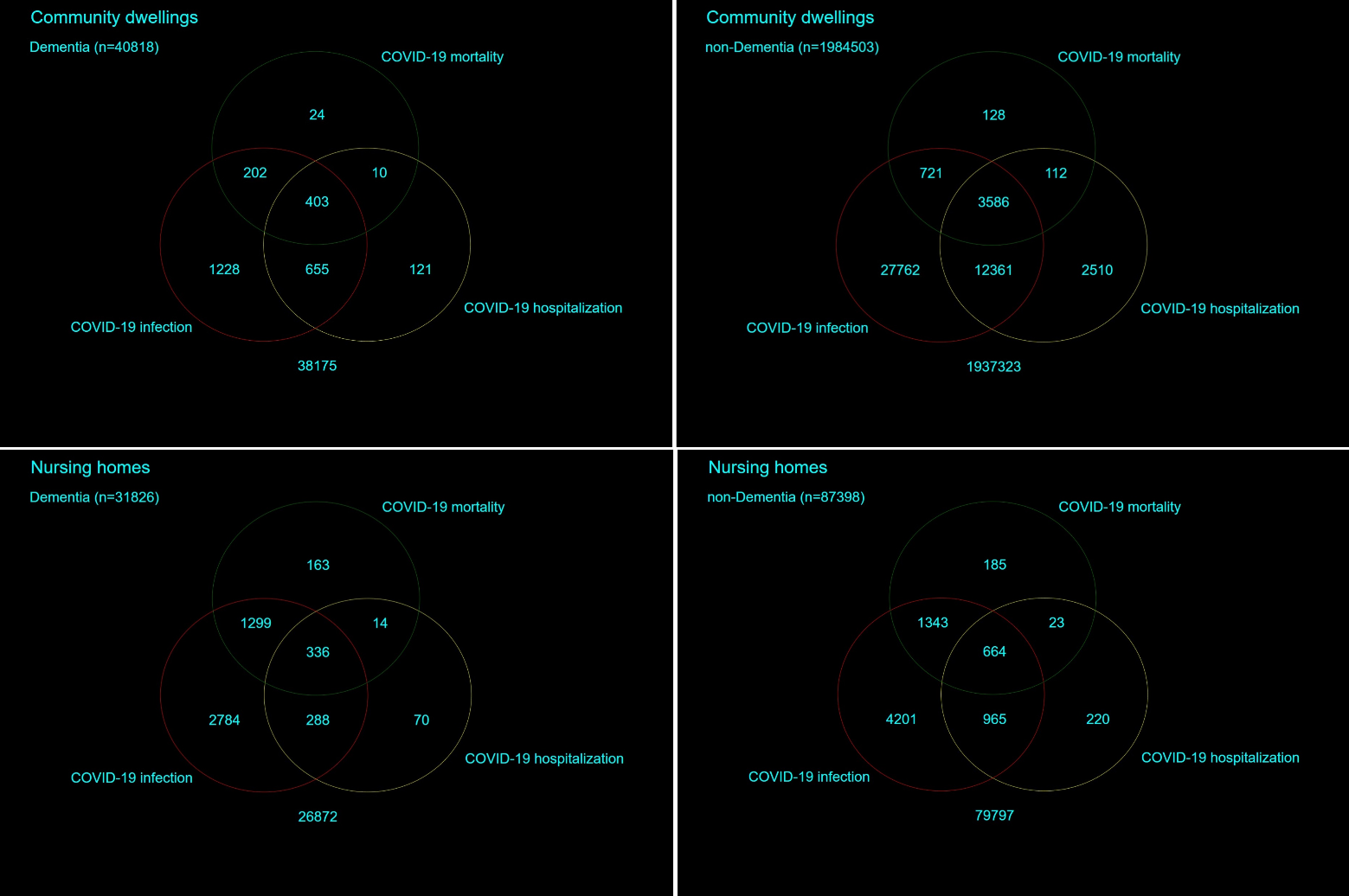


## References for Supplements

1. Benchimol EI, Smeeth L, Guttmann A, Harron K, Moher D, Petersen I, et al. The REporting of studies Conducted using Observational Routinely-collected health Data (RECORD) statement. PLoS Med. 2015;12(10):e1001885.

2. Ludvigsson JF, Almqvist C, Bonamy AK, Ljung R, Michaëlsson K, Neovius M, et al. Registers of the Swedish total population and their use in medical research. European journal of epidemiology. 2016;31(2):125-36.

3. Ludvigsson JF, Svedberg P, Olen O, Bruze G, Neovius M. The longitudinal integrated database for health insurance and labour market studies (LISA) and its use in medical research. Eur J Epidemiol. 2019;34(4):423-37.

4. Ludvigsson JF, Andersson E, Ekbom A, Feychting M, Kim JL, Reuterwall C, et al. External review and validation of the Swedish national inpatient register. BMC Public Health. 2011;11:450.

5. Wallerstedt SM, Wettermark B, Hoffmann M. The First Decade with the Swedish Prescribed Drug Register - A Systematic Review of the Output in the Scientific Literature. Basic Clin Pharmacol Toxicol. 2016;119(5):464-9.

6. Wettermark B, Hammar N, Fored CM, Leimanis A, Otterblad Olausson P, Bergman U, et al. The new Swedish Prescribed Drug Register--opportunities for pharmacoepidemiological research and experience from the first six months. Pharmacoepidemiol Drug Saf. 2007;16(7):726-35.

7. Brooke HL, Talbäck M, Hörnblad J, Johansson LA, Ludvigsson JF, Druid H, et al. The Swedish cause of death register. Eur J Epidemiol. 2017;32(9):765-73.

8. Meyer AC, Sandström G, Modig K. Nationwide data on home care and care home residence: presentation of the Swedish Social Service Register, its content and coverage. 2022;50(7):946-58.

9. Rolfhamre P, Janson A, Arneborn M, Ekdahl K. SmiNet-2: Description of an internet-based surveillance system for communicable diseases in Sweden. Euro Surveill. 2006;11(5):15-6.

10. World Health O. International statistical classification of diseases and related health problems. 10th revision, Fifth edition, 2016 ed. Geneva: World Health Organization; 2015 2015.

11. Calderón-Larrañaga A, Vetrano DL, Onder G, Gimeno-Feliu LA, Coscollar-Santaliestra C, Carfí A, et al. Assessing and Measuring Chronic Multimorbidity in the Older Population: A Proposal for Its Operationalization. J Gerontol A Biol Sci Med Sci. 2017;72(10):1417-23.

12. World Health Organization Collaborating Centre for Drug Statistics Methodology. ATC/DDD Index 2021 Norway2021 (updated October 22, 2021; cited 2021 October 22). Available from: <https://www.whocc.no/atc_ddd_index/>.

13. Gilbert T, Neuburger J, Kraindler J, Keeble E, Smith P, Ariti C, et al. Development and validation of a Hospital Frailty Risk Score focusing on older people in acute care settings using electronic hospital records: an observational study. Lancet. 2018;391(10132):1775-82.
